# Crosstalk Between Trophoblast and Macrophage at the Maternal-Fetal Interface: Current Status and Future Perspectives

**DOI:** 10.3389/fimmu.2021.758281

**Published:** 2021-10-21

**Authors:** Jinli Ding, Yan Zhang, Xiaopeng Cai, Lianghui Diao, Chaogang Yang, Jing Yang

**Affiliations:** ^1^ Reproductive Medical Center, Renmin Hospital of Wuhan University, Hubei Clinic Research Center for Assisted Reproductive Technology and Embryonic Development, Wuhan, China; ^2^ Department of Clinical Laboratory, Renmin Hospital of Wuhan University, Wuhan, China; ^3^ Department of Gastrointestinal Surgery, The Clinical Medical Research Center of Peritoneal Cancer of Wuhan, Hubei Key Laboratory of Tumor Biological Behaviors, Hubei Cancer Clinical Study Center, Zhongnan Hospital of Wuhan University, Wuhan, China; ^4^ Shenzhen Key Laboratory of Reproductive Immunology for Periimplantation, Shenzhen Zhongshan Institute for Reproduction and Genetics, Shenzhen Zhongshan Urology Hospital, Shenzhen, China

**Keywords:** maternal-fetal interface, trophoblast, macrophage, pregnancy, immune tolerance

## Abstract

The immune tolerance microenvironment is crucial for the establishment and maintenance of pregnancy at the maternal-fetal interface. The maternal-fetal interface is a complex system containing various cells, including lymphocytes, decidual stromal cells, and trophoblasts. Macrophages are the second-largest leukocytes at the maternal-fetal interface, which has been demonstrated to play essential roles in remodeling spiral arteries, maintaining maternal-fetal immune tolerance, and regulating trophoblast’s biological behaviors. Many researchers, including us, have conducted a series of studies on the crosstalk between macrophages and trophoblasts at the maternal-fetal interface: on the one hand, macrophages can affect the invasion and migration of trophoblasts; on the other hand, trophoblasts can regulate macrophage polarization and influence the state of the maternal-fetal immune microenvironment. In this review, we systemically introduce the functions of macrophages and trophoblasts and the cell-cell interaction between them for the establishment and maintenance of pregnancy. Advances in this area will further accelerate the basic research and clinical translation of reproductive medicine.

## Introduction

The establishment and maintenance of normal pregnancy is a complex process involving multiple cells and various molecules. Among them, the precise regulation at the maternal-fetal interface plays a pivotal role. The maternal-fetal interface comprises decidual immune cells, stromal cells, and trophoblasts, characterized by maintaining the defense against possible pathogens and immune tolerance to the allogeneic fetus (1). Increasing evidence demonstrates that interaction among these cellular components can influence the maternal-fetal interactive dialogue, thereby participating in the regulation of the pregnancy program (1). Therefore, exploring the cell-cell crosstalk at the maternal-fetal interface will help us further understand pregnancy’s physiological and pathological processes.

During early pregnancy, 30-40% of the decidual cells are leukocytes, including macrophages, natural killer (NK) cells, B cells, T cells, and dendritic cells (2). Macrophages, as antigen-presenting cells, comprise 20-30% of the leukocytes at the maternal-fetal interface (3), which participate in the complex regulation at the maternal-fetal interface by regulating the secrete cytokines, phagocytosis, and immune balance (4). Besides their effects on vascular remodeling, macrophages are actively associated with trophoblasts invasion and pregnancy maintenance (5). Macrophages could be divided into classically activated (M1) and alternatively activated (M2) subtypes based on their cytokine production and function (5). The polarization states of decidual macrophages undergo dynamic changes to changing microenvironment at different gestational ages, adjusting to the different stages of fetal development. It has been demonstrated that aberrantly activated macrophages at the maternal-fetal interface may be closely related to various pregnancy complications, including miscarriage, preeclampsia, preterm birth, fetal growth restriction, or demise (6–9). Accumulating evidence indicates that the polarization of macrophages is regulated by a variety of cytokines, chemokines, sex hormones, and cell-cell interactions at the maternal-fetal interface, among which trophoblasts exert pivotal roles (4, 10, 11). Trophoblasts are also important elements at the maternal-fetal interface, which invade the maternal myometrium and direct contact with maternal decidual stromal cells (12). Sufficient trophoblasts invasion could facilitate maternal spiral artery remodeling and placental blood flow, providing a favorable embryo implantation environment (12). Previous studies showed that the maternal microenvironment temporally and spatially controls trophoblast invasion at the maternal-fetal interface, including arteries, glands, decidual NK cells, macrophages and stromal cells (13). Trophoblasts can respond to various mediators secreted by polarized macrophages to regulate their biological behaviors (6, 14–16), while soluble cytokines produced by trophoblasts could induce macrophages polarization (17–19), involving in the regulation of normal pregnancy. Previously, our group conducted a series of studies on the crosstalk between macrophages and trophoblasts at the maternal-fetal interface: on the one hand, trophoblasts can regulate the polarization of the M2 macrophages by secreting IL-6 (20); on the other hand, M2 macrophages can promote the invasion and migration of trophoblasts by secreting G-CSF, thereby regulating the establishment and maintenance of normal pregnancy (21). In addition, macrophages could also secrete exosomes and deliver miRNAs to target and regulate the invasion and migration capabilities of trophoblasts, thereby participating in the occurrence of recurrent spontaneous abortion (RSA) (22). Although, the understanding of the interaction between trophoblasts and macrophages at the maternal-fetal interface is still insufficient and need to be further explored.

In the present review, we aimed to summarize the crosstalk between macrophages and trophoblasts at the maternal-fetal interface and the functions in normal pregnancy and pregnancy complications, providing the latest progress in the field and bringing more profound ideas to researches.

## The Role of Macrophages at the Maternal-Interface

Generally, macrophages are classified as M1 and M2 phenotypes based on the different function and cytokine production, but in fact, there is a full spectrum of macrophage between M1 and M2 (2). Therefore, macrophages are highly plastic and heterogeneous, here we just focus on M1 and M2 as typical subtypes to explore their functions. M1 could be induced by toll-like receptor ligands, bacterial lipopolysaccharide (LPS) or other cytokines (including TNF-α, IFN-γ, and GM-CSF), which produces reactive oxygen species and high level of IL-1β, IL-6, IL-12 and IL-23 (3, 4). M2 can be polarized by cytokines like IL-4, IL-13, IL-10, immune complexes, LPS and M-CSF, producing high levels of TGF-β and IL-10. M2 plays important roles in clearing apoptotic cells, tissue repair and remodeling (4–6). M2 can be further divided into four subtypes, including M2a, M2b, M2c and M2d. M2a-Mφ could be induced by IL-4 and IL-13, which expresses high level of CD206, TGF-β, insulin-like growth factor (IGF) and IL-1 receptor (IL-R) (7). M2b-Mφ can be induced by immune complexes plus LPS or IL-1β, which expresses and secretes proinflammatory cytokine including TNF-α, IL-6 and IL-1β, and anti-inflammatory cytokine IL-10 (8). By contrast, M2c-Mφ is induced by IL-10 and releases high levels of IL-10 and TGF-β, exerting the function of phagocytosis of apoptotic cells (4). In addition, M2d-Mφ, also known as tumor-associated macrophages, can be induced by IL-6, or A2 adenosine receptor plus toll-like receptor ligands, mainly characterized by high expression level of TGF-β, IL-10 and vascular endothelial growth factor (VEGF), playing essential roles in cancer metastasis and angiogenesis (9).

In the context of reproduction, macrophages are present in the endometrium, decidua and placenta, which comprise 20-30% of the leukocytes. Affected by estrogen and progesterone, the number of macrophages fluctuates during the menstrual cycle (10, 11). During the embryo implantation window, macrophages exhibit M1 phenotype. As trophoblasts implant and invade the endometrium, macrophages transform into a mixed M1/M2 type, which lasts to the first trimester and the early stage of the second trimester (12). At the second trimester, macrophages are polarized to the M2 phenotype to prevent rejection of the fetus by the maternal system and maintain fetal growth until delivery (13). At the time of delivery, the macrophages appear to be M1 subtype (14). Although increasing evidence has demonstrated the roles and distribution of decidual macrophages (DMs), the classification and subtypes of DMs are still controversial. Based on the expression level of CD11c and CD209, DMs are divided into CD11c^low^ and CD11c^high^ subgroups (15), or CD209^−^ and CD209^high^ macrophages (16). Jiang et al. divided DMs into three subtypes depending on the expression of CCR2 and CD11c, including CCR2^−^CD11c^low^, CCR2^−^CD11c^high^ and CCR2^+^CD11c^high^ (17). Evidence has demonstrated that M2-Mφ dominates at the maternal-fetal interface, maintaining the immune-suppressive environment toward the fetus (18).

Increasing studies demonstrate that macrophages play essential roles in the establishment and maintenance of normal pregnancy, including spiral artery remodel, apoptotic cell phagocytosis and trophoblasts functions (19–21). The polarization balance between M1-Mφ and M2-Mφ is important for various processes of normal pregnancy. Conversely, the dysregulated macrophages polarization is associated with a variety of pregnancy complications, including RSA, preeclampsia (PE), fetal growth restriction and preterm labor (14, 22–25). The increased number of M1-Mφ and the decreased of M2-Mφ at the maternal-interface is related with RSA (23) and PE (19), accompanied by a decrease in anti-inflammatory cytokines and an increase in pro-inflammatory cytokines. In addition, DMs differentiated into M1-Mφ is related to spontaneous preterm labor (14). Animal experiments demonstrate that induction of M1 macrophages polarization increases embryo resorption (26), while suppressing M1 macrophages polarization alleviates the opposite effect (27). Moreover, macrophages have high plasticity, and their polarization and function are affected by the surrounding environment (4). It has been reported that hormones, chemokines, cytokines, growth factors, and the crosstalk between macrophages and related cells are involved in the regulation (28). In addition, there is growing evidence confirming the microbiome colonization at the maternal-fetal interface (29, 30). Compounds originating from the microbiome might bind to aryl hydrocarbon receptor (AhR), which is widely expressed in adaptive and innate immune cells including macrophages (31). Indeed, AhR plays an important role in regulating macrophage responsiveness and the expression of AhR in macrophage is essential for homeostasis and inflammatory responses (31, 32).

## The Role of Trophoblasts at the Maternal-Interface

In the human placenta, there are three trophoblast subpopulations: the cytotrophoblasts (CTBs), syncytiotrophoblasts (STBs) and extravillous cytotrophoblasts (EVTs) (33). After embryo implantation, the outermost layer of the blastocysts transforms into mononuclear CTBs. Proliferative CTBs make up the primary villi, then the primary villi further differentiates into secondary mesenchymal villi and mature tertiary villi. Cell fusion of villous CTBs generates STBs, which plays important roles in transport of oxygen and nutrients, production of pregnancy hormones and clearance of fetal waste products (34). STBs of the floating villi represent the transport units of the human placenta, while anchoring villi transforms into another trophoblast type, namely EVTs (35). EVTs are migratory and invasive trophoblasts, which can migrate into decidual lymphatics, veins and glands, participating in placental embedment and fetal development (36–38). Defects in spiral artery remodeling, and failure in the interactions of EVTs with uterine vessels might lead to pregnancy complications, as lower numbers of EVTs in lymphatic and venous vessels is found in RSA (37). Invasive EVTs remodel the maternal spiral arteries, ensuring an adequate maternal blood supply for normal fetal growth and development. The process reduces maternal blood pressure and flow velocity, meeting the increased uteroplacental perfusion required by the developing fetus (39). The arteries remain narrow at the openings, and blood enters the intervillous space at a higher velocity, which leads to damaged villi structure and impaired placental function (40). Trophoblast invasion is temporally and spatially controlled by the maternal uterus environment, including arteries, glands, decidual NK cells, macrophages and stromal cells. Insufficient EVTs invasion has been reported to be related with pregnancy complications including RSA, PE, stillbirth and fetal intrauterine growth restriction (41–44), while excessive invasion also might lead to pathological condition such as placenta accrete (45). In addition, trophoblasts serve as a member of innate immune system at the maternal-fetal interface, as trophoblasts could recognize and respond to viral and bacterial products (46), and trophoblasts might integrate microbial-derived signals *via* alternative pathways mediating the response to TLRs or epigenetic modifications (47, 48). Therefore, exploring the factors affecting trophoblasts functions is of great significance for further understanding of the normal pregnancy process and the pathogenesis of pregnancy complications.

## Crosstalk Between Trophoblasts and Macrophages at the Maternal-Fetal Interface

**Figure 1:**
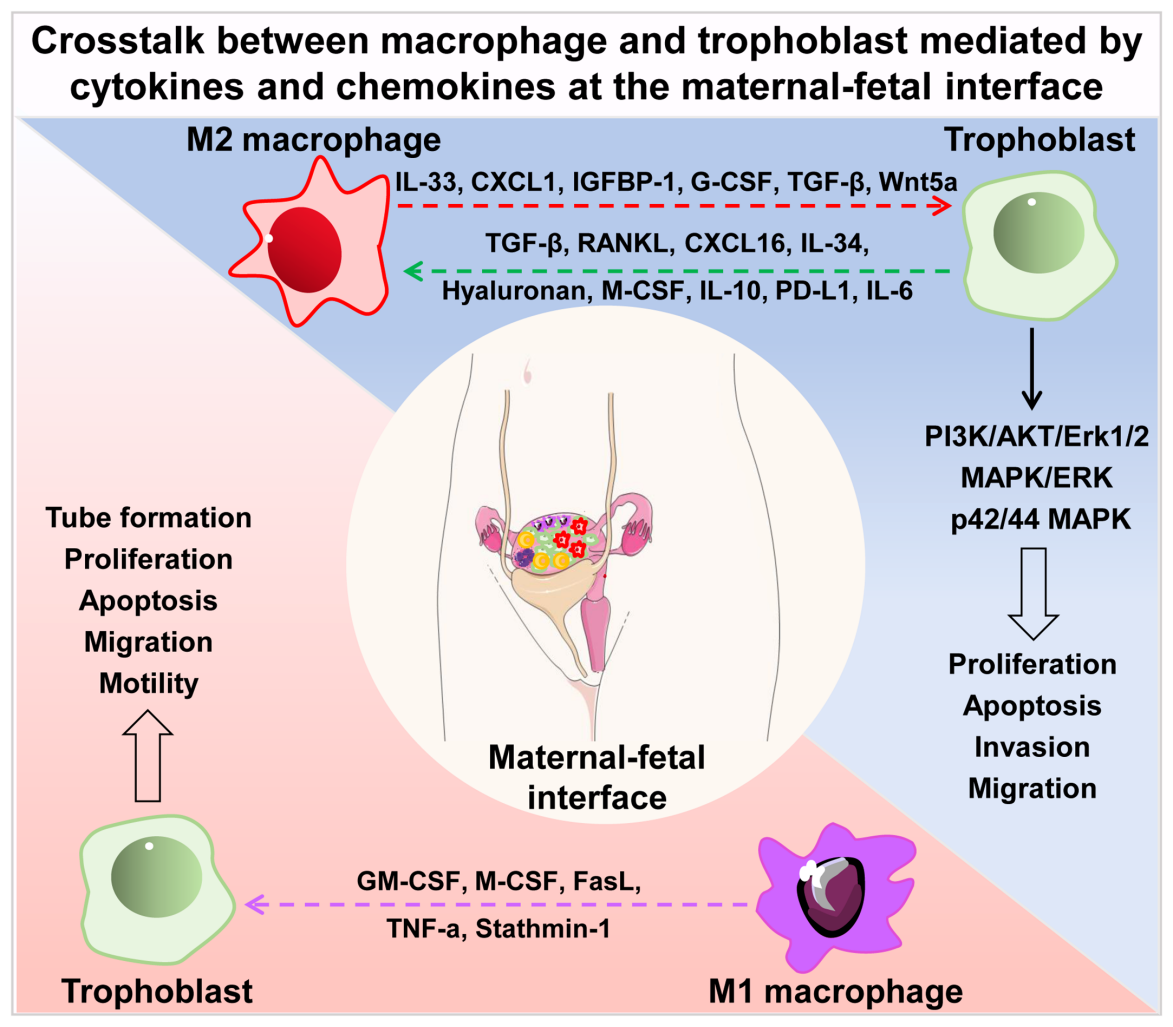
Crosstalk between macrophage and trophoblast mediated by cytokines and chemokines at the maternal-fetal interface. Cytokines and chemokines secreted by M2 macrophages (IL-33, CXCL1, IGFBP1, G-CSF, TGF-β, Wnt5a) regulate the proliferation, apoptosis, invasion and migration of trophoblast *via* PI3K/AKT/Erk1/2, MAPK/ERK and p42/44 MAPK signal pathways. Trophoblast- derived secretory factors (TGF-β, RANKL, CXCL16, IL-34, hyaluronan, M-CSF, IL-10, PD-L1, IL-6) drive M2 macrophage polarization in turn. Soluble molecules secreted by M1 macrophages (GM-CSF, M-CSF, FasL, TNF-a, Stathmin-1) influence the tube formation, proliferation, apoptosis, migration and motility of trophoblast. IL, interleukin; CXCL, chemokine (C-X-C motif) ligand; IGFBP1, insulin-like growth factor-binding protein-1; G-CSF, granulocyte colony stimulating factor; TGF-β, transforming growth factor β; RANKL, receptor activator of NF-κB ligand; M-CSF, macrophage colony stimulating factor; PD-L1, programmed cell death 1 ligand 1; GM-CSF, granulocyte-macrophage colony stimulating factor; FasL, factor associated suicide ligand; TNF-α, tumor necrosis factor α.

In this section, we summarized the interaction between trophoblasts and macrophages, and the role in the establishment and maintenance of normal pregnancy and pathological pregnancy (**Figure 1**).

### The Effect of Macrophages on Trophoblasts Functions

Precise regulation of the proliferation, apoptosis, migration and invasion of trophoblasts are essential for the establishment and maintenance of pregnancy. In local microenvironment, abundant DMs are observed at the implantation site and the invasive front of EVTs (49). Thence, paracrine activity of macrophages exerts an important role in biological function of trophoblasts (15). Results from rhesus monkey indicated that the growth and differentiation of embryonic trophoblasts were regulated by macrophages (50). Here, we will elaborate on the effect of macrophages on proliferation, apoptosis, migration and invasion of trophoblasts in recent years.

#### Proliferation and Apoptosis

Trophoblast apoptosis may induce inflammatory events that might initiate trophoblasts dysfunction, leading to pregnancy complications (23, 51). Macrophages with different polarization statues have different effects on the motility and tube formation of trophoblasts: M1 macrophage exerted an inhibitory role, while M2 macrophages had the opposite effect (52). The Fas/FasL system is one of the major apoptotic pathways in tissues and cells. Results from our and others’ group demonstrated that macrophages could induce trophoblasts apoptosis *via* FasL (23, 53). In addition, IL-33, as a member of the IL-1 family, has been demonstrated to promote the proliferation of cell column trophoblasts, villous cytotrophoblasts and primary trophoblasts, which was mediated by PI3K/AKT and MAPK/ERK signaling (54). Moreover, previous report also showed that macrophages could promote trophoblasts proliferation by secreting Wnt5a *via* p42/44 MAPK pathway (55). Meanwhile, decidual cells stimulated with pro-inflammatory cytokines including TNF-α and IL-1β significantly strengthened the promotion effect of macrophage-induced caspase-dependent trophoblasts apoptosis *via* GM-CSF and M-CSF (56).

#### Invasion and Migration

The regulatory effects of macrophages on the invasion and migration of trophoblasts are depend on their polarization states. Renaud et al. demonstrated that nonactivated macrophages had no effect on trophoblasts invasion, while macrophages activated by LPS inhibited the ability of trophoblasts to invade through extracellular matrix *in vitro* (57), and the effect could be reversed by IL-10 (58). In addition, M1 macrophage-derived TNF-a reduced the expression of stathmin-1 in trophoblasts, regulating the proliferation and invasion ability, *via* E-cadherin/β-catenin pathway (44). TGF-β is a soluble mediator produced by M2 macrophages, and our recent research shows that TGF-β secreted by M2 macrophage could induce trophoblast migration and invasion (59). Human leukocyte antigen G5 (HLA-G5) has been reported to play essential roles in immune tolerance during normal pregnancy. HLA-G5 is not expressed in the DMs, while secreted HLA-G5 could be detected in the exosomes of primary mononuclear cytotrophoblast cells and placental explants of first trimester (60). It has been confirmed that trophoblasts-derived HLA-G5 could difference monocytes into M2-Mφ, which in turn promotes the invasion ability of trophoblasts *via* secreting CXCL1 (61). Glycodelin-A is a glycoprotein abundantly present in the decidua, playing an important role in immune cell regulation at the maternal-fetal interface (62). Glycodelin-A could induce the differentiation of monocytes towards DMs-like phenotype by binding to Siglec-7, which promotes trophoblast invasion *via* the production of insulin-like growth factor-binding protein 1 (IGFBP-1) (63), a regulator of trophoblast functions and angiogenesis during pregnancy (64). Medroxyprogesterone is widely used for the treatment of endometrium cancer, abnormal uterine bleeding and secondary amenorrhea (65). Recently, it has been proved that medroxyprogesterone could drive monocyte differentiation toward M2 macrophages *via* ERK phosphorylation, which promoted the invasion activity of trophoblasts and the decidualization of endometrial stromal cells (66). Granulocyte colony-stimulating factor (G-CSF) is secreted by multiple cells including macrophages (67, 68) and placental villous trophoblasts (69). Increasing evidence demonstrates the therapeutic effect of G-CSF in RSA, repeated implantation failure, luteinized unruptured follicle syndrome and women with thin endometrium (70–72). Previously, by using a co-cultured model, our study showed that trophoblasts-derived IL-6 promoted M2 macrophage polarization (73), which in turn induced the epithelial-mesenchymal transition of trophoblasts, thereby promoting migration and invasion though secreting G-CSF to activate PI3K/AKT/Erk1/2 signaling pathway (68).

### The Effect of Trophoblasts on Macrophages Polarization

Macrophage polarization is a complex process and plastic to the environment surrounding or in contact with them, which is involved in multiple factors including cytokines, chemokines, growth factors, etc. Among which, cell-cell interaction exerts a vital role. DMs and EVTs are accumulated at the implantation site, and factors secreted by trophoblasts play important roles in regulating the differentiation and function of macrophages. However, the factors involved in this process has not been elucidated. In this part, we mainly focus on the effect of trophoblasts on the differentiation and function of macrophages.

Trophoblast debris have been reported to modulate the expression of cytokines in macrophages, upregulating the expression of programmed death-1 ligand 1 (PD-L1), indoleamine 2,3-dioxygenase (IDO), and anti-inflammatory cytokines including IL-1Ra, IL-6, and IL-10, while reducing the expression of costimulatory molecules (B7H3, CD40, CD80 and CD86), MHC-II molecules, IL-8 receptors, inter-cellular adhesion molecule 1 (ICAM-1), monocyte chemoattractant protein-1 (MCP-1), and pro-inflammatory cytokines including IL-8, IL-1β, and IL-12p70 (74). Aldo et al. reported that trophoblast-derived TGF-β could induce monocyte differentiation into CD14^+^/CD16^+^ macrophages, accompanied by high secretion levels of IL-1β, IL-10, and interferon-inducible protein-10 (IP-10), and increased phagocytosis capacity (75). By secreting IL-10 and M-CSF, trophoblasts could induce M2 macrophages polarization, sharing the phenotype of CD163^+^CD206^+^CD209^+^ and producing high level of CCL18 and IL-10 (76). IL-34 is a second ligand for the M-CSF receptor, which is involved in the maintenance and development of other macrophage subsets, such as osteoclasts (77) and Langerhans cells (78). IL-34 is produced by both decidual stromal cells and trophoblasts, which could polarize monocytes into M2 macrophages with the similar cytokine secretion pattern with DMs (79). Recently, our group has demonstrated that trophoblasts-derived IL-6 could induce M2 macrophages polarization by activating STAT3 signal pathway, with high expression of CD206, CCL18, IL-10 and TGF-β (73). In addition, CXCL16 derived from trophoblasts differenced monocytes into M2 macrophages by binding to CXCR6, which exhibited decreased IL-15 production, facilitating the inactivation of NK cells and restricting the cytotoxicity of NK cells (80).

Moreover, it has been demonstrated that PD-1/PD-L1 pathway exerts an important role in regulation of immune cell homeostasis, especially macrophage polarization (26) and T cells activation (81). PD-L1 was reported to be expressed in the trophoblasts of normal placenta, and decreased expression of PD-L1 was confirmed in pregnancy complications such as RSA (26). PD-L1 expression/secretion of trophoblasts could be promoted by IFN-β, inducing PD-1/PD-L1-mediated M2 macrophage polarization (82). Similar to PD-L1, trophoblast-derived HLA-G5 could drive M2 macrophages polarization manifested by increased IDO 1 and IL-6 expression (61). In addition to cytokines and chemokines, the regulatory roles of other factors have been reported. Hyaluronan (HA) is an unbranched polymer and widely present in the extracellular matrix of mammalian tissues. Decreased expression of HA was detected in villi of miscarriage patients (83). Wang et al. demonstrated that HA derived from trophoblasts could induce M2 macrophages polarization by interacting with CD44 *via* PI3K/AKT-STAT3/STAT-6 pathway (84). It has been indicated that HA are divided into four subtypes depending on the molecular weight, high molecular weight HA, medium molecular weight HA, low molecular weight HA and HA oligomers (85). HA with different molecular weight might have different effects on macrophage polarization, low molecular weight HA drives M1 macrophage polarization featured with increased expression of CD80, iNOS2, TNF-α, NO and IL-12β, while high molecular weight HA induces M2 macrophage polarization charactered by increased expression of Arg1, MRC1, IL-10 and enhanced arginase activity (86, 87). As mentioned above, AhR is expressed in macrophages and plays an important role in regulating macrophage responsiveness (31, 32). Indeed, the expression of AhR in trophoblasts has been confirmed in 2018 (88). Therefore, AhR might play a role in the pregnancy program *via* regulating the crosstalk between trophoblasts and macrophages. What’s more, lower expression of placental AhR was found in unexplained RSA women, and the activation of AhR might impair the proliferation and migration of trophoblasts (89).

Receptor activator of NF-κB ligand (RANKL) and its tumor necrosis factor (TNF)-family receptor RANK exert important roles in lymph node formation, bone remodeling, thymic microenvironment establishment, and mammary gland development during pregnancy. RANKL derived from trophoblasts could drive macrophage polarization to M2 subtype *via* activating AKT/STAT6-Jmjd3/IRF4 signaling pathway, and decreased RANKL in trophoblasts and RANK on DMs was observed in patients suffered from miscarriage (90). By using knockout mice, the authors further demonstrated that RANKL^−/−^ mice displayed macrophage dysfunction and increased fetal loss, while adoptive transfer of RANK^+^ macrophages could relieve the fetal loss induced by macrophage depletion (90).

Altogether, results from the above studies confirm the role of trophoblasts on M2 macrophage polarization, which were based on trophoblasts from healthy pregnancy women or trophoblast cell lines. However, it is not known whether these roles were maintained in a pathological condition, as trophoblast dysfunction was related to pregnancy complications such as unexplained RSA. A previous study from an *ex vivo* model demonstrated that trophoblasts from unexplained RSA could promote an M2-like phenotype, indicating that trophoblasts from unexplained RSA had similar functional and proteomic properties with trophoblasts from healthy women (91). Cluster of differentiation 74 (CD74) was a high-affinity binding protein for the inflammatory cytokine macrophage migration inhibitory factor, which was released by trophoblasts to regulate monocyte activity (92). Przybyl et al. demonstrated that the adhesion of macrophages lacking CD74 to trophoblasts was decreased, exhibiting the pro-inflammatory phenotype when co-cultured with trophoblasts (24).

## The Role of Extracellular Vesicles in Crosstalk Between Macrophages and Trophoblasts at the Maternal-Fetal Interface

**Figure 2:**
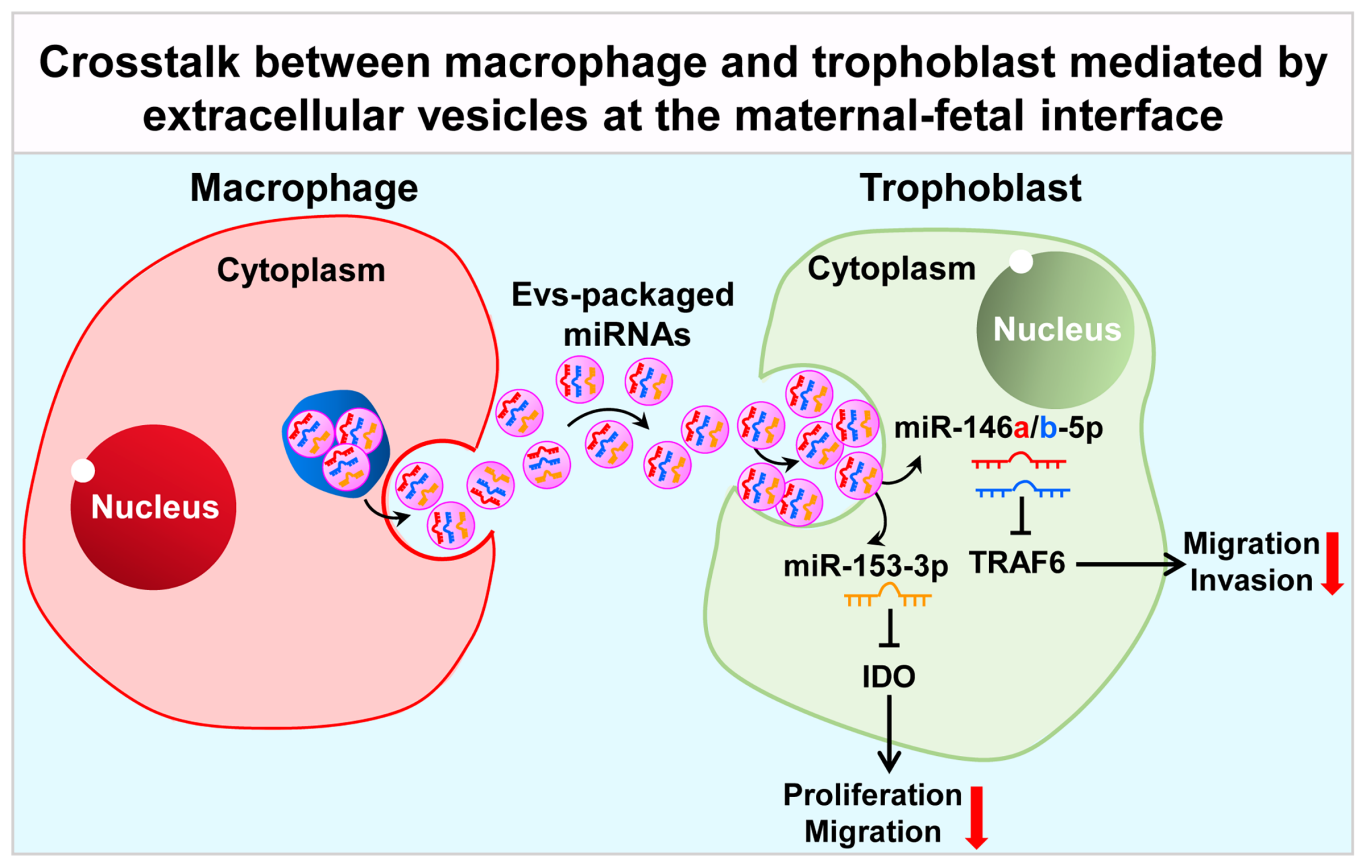
Crosstalk between macrophage and trophoblast mediated by extracellular vesicles at the maternal-fetal interface. Macrophage-derived extracellular vesicles affect the expression of TRAF6 and IDO in trophoblast by delivering miR-146a-5p, miR-146b-5p and miR-153-3p, thereby affecting their invasion and migration capabilities, participating in the pathological process of RSA. TRAF6, TNF receptor associated factor 6; IDO, indoleamine 2, 3-dioxygenase.

In addition to soluble factors, extracellular vesicles (EVs) are also demonstrated to be another important medium in communication network at the maternal-fetal interface in recent years (93). EVs, including microvesicles, exosomes and apoptotic bodies, are intricately involved in cell-cell communication, which are released by various types of cells, including macrophages and trophoblasts. EVs contain abundant molecules like lipids, proteins, DNA, mRNAs, long non-coding RNAs (LncRNA) and miRNAs, which are delivered to target cells (94). EVs could be detected in multiple biofluids including milk, urine, blood, saliva and amniotic fluid (95). EVs concentration in circulation is increased in pregnant women, with 5-folds higher than non-pregnant women (96), which could be detected in maternal plasma at 6 weeks of gestational age (97). Trophoblasts-released EVs are taken up by cells of maternal vascular and immune systems, regulating the maternal physiological system to adapt to the pregnancy process (93). Placental EVs has been proven to exert an important role in maintaining maternal-fetal immune tolerance *via* repression of modulatory signals in the maternal immune system and suppression of natural killer cells and T-lymphocyte activation (98). The release of EVs by EVTs has been confirmed as HLA-G^+^ EVs were detected in the maternal circulation during pregnancy (99). In this section, we mainly summarized the latest progress about the role of EVs in crosstalk between macrophages and trophoblasts at the maternal-fetal interface (**Figure 2**).

In fact, little is known about EVs-mediated crosstalk between macrophages and trophoblasts up to now. Atay et al. firstly reported that trophoblast-derived exosomes could recruit and educate monocytes to produce G-CSF, granulocyte/monocyte colony-stimulating factor (GM-CSF), IL-1β, IL-6, TNF-α and Serpin-E1, in a cell-contact-independent manner, which were necessary for embryo implantation, stromal remodeling and angiogenesis (100). These authors further addressed the mechanism that IL-1β induction by exosome-associated fibronectin (101). EVs released by macrophages in turn modulate trophoblast’s function. Holder et al. reported that macrophages-derived exosomes were internalized by human placenta in a time- and dose-dependent manner, *via* clathrin-dependent endocytosis, inducing the release of proinflammatory cytokines, including IL-6, IL-8 and IL-10 (102). EVs exert biological functions mainly through the biologically molecules they conduct. For example, EVs released from DMs could deliver miR-153-3p to target IDO, inhibiting trophoblasts proliferation and migration *via* STAT3 pathway and contributing to occurrence of unexplained RSA (103). Recently, our group reported that EVs derived from M1-Mφ could deliver miR-146a-5p and miR-146b-5p to target TNF receptor associated factor 6 (TRAF6), suppressing trophoblast migration and invasion and contributing to the development of RSA (104). However, more evidence is needed to confirm the role of EVs in the establishment and maintenance of pregnancy.

## Conclusions and Perspectives

Taken together, current evidence suggests that trophoblasts and macrophages can establish extensive connections at the maternal-fetal interface and thus participate in regulating the physiological and pathological processes of pregnancy. Polarized macrophages can influence the biological behavior (including proliferation, apoptosis, invasion and migration) through the secretion of various cytokines and chemokines. Reciprocally, trophoblasts can also regulate the polarization state of macrophages through a variety of mechanisms, thus affecting the establishment and maintenance of immune tolerance microenvironment at the maternal-fetal interface.

In spite of this, the interaction between trophoblasts and macrophages still needs further study: 1) As above presented, the crosstalk reported for now between trophoblasts and macrophages are mainly mediated by cytokines and chemokines. EVs, as an emerging intercellular communication medium, have been gradually proven to exert role in mediating the mutual communication between cells. However, the role and potential mechanisms of EVs in the crosstalk between macrophages and trophoblasts at the maternal-fetal interface is rarely studied, which is an emerging field that needs to be explored urgently. 2) What’ more, in addition to macrophages, there are also immune cells including T cells, NK cells and dendritic cells at the maternal-fetal interface, which also play important roles in regulating the biological behavior of trophoblasts. Then, whether other cells may also participate in the interaction between macrophages and trophoblasts, thereby establishing three-cell or multi-cell communication, and then participating in the establishment and maintenance of pregnancy, is also worth exploring in future research. 3) Additionally, as most of the current researches are based on cell lines, transforming the interaction of macrophages and trophoblasts into clinical research is also a direction worth studying.

## Author Contributions

CY and JY designed the review. JD, YZ, and XC drafted the manuscript and prepared the figures. LD helped to modify the manuscript. All authors contributed to the article and approved the submitted version.

## Funding

This work was supported by grants from the National Key Research and Development Program of China (No. 2018YFC1002804), the National Natural Science Foundation of China (No. 82101749, 81771662, 81971356 and 81801540), the Fundamental Research Funds for the Central Universities (No. 2042021kf0082 and 2042021kf0143), Zhongnan Hospital of Wuhan University, Excellent Doctor Fund Project (No. ZNYB2020002), and Technology and Innovation Seed Found (No. CXPY2020025).

## Conflict of Interest

The authors declare that the research was conducted in the absence of any commercial or financial relationships that could be construed as a potential conflict of interest.

## Publisher’s Note

All claims expressed in this article are solely those of the authors and do not necessarily represent those of their affiliated organizations, or those of the publisher, the editors and the reviewers. Any product that may be evaluated in this article, or claim that may be made by its manufacturer, is not guaranteed or endorsed by the publisher.
